# A Multimodal Sensing Device for Simultaneous Measurement of Dissolved Oxygen and Hydrogen Ions by Monolithic Integration of FET-Based Sensors

**DOI:** 10.3390/s22176669

**Published:** 2022-09-03

**Authors:** Toshihiko Noda, Sylvia Mei Lin Loo, Yoshiko Noda, Daisuke Akai, Takeshi Hizawa, Yong-Joon Choi, Kazuhiro Takahashi, Kazuaki Sawada

**Affiliations:** 1 Electronics Inspired-Interdisciplinary Research Institute (EIIRIS), Toyohashi University of Technology, Toyohashi 441-8580, Japan; 2 Department of Electrical and Electronic Information Engineering, Toyohashi University of Technology, Toyohashi 441-8580, Japan; 3 Office for Technical Support Services, Toyohashi University of Technology, Toyohashi 441-8580, Japan

**Keywords:** dissolved oxygen, hydrogen ion, multimodal sensing, ISFET, solid-state sensor, potentiometric sensing

## Abstract

We examined the possibility of measuring dissolved oxygen by using a potentiometric solid-state semiconductor sensor. Thin films of tin (IV) oxide (SnO_2_) are widely used in oxygen gas sensors. However, their ability to detect dissolved oxygen (DO) in solutions is still unknown. In this paper, we present a method for investigating the dissolved oxygen-sensing properties of SnO_2_ thin films in solutions by fabricating a SnO_2_-gate field-effect transistor (FET). A similarly structured hydrogen ion-sensitive silicon nitride (Si_3_N_4_)-gate FET was fabricated using the same method. The transfer characteristics and sensitivities were experimentally obtained and compared. The transfer characteristics of the FET show a shift in threshold voltage in response to a decrease in DO concentration. The SnO_2_-gate FET exhibited a sensitivity of 4 mV/ppm, whereas the Si_3_N_4_-gate FET showed no response to DO. Although the SnO_2_-gate FET responds to pH changes in the solution, this sensitivity issue can be eliminated by using a Si_3_N_4_-gate FET, which is capable of selectively sensing hydrogen ions without DO sensitivity. The experimental results indicate the promising properties of SnO_2_ thin films for multimodal sensing applications.

## 1. Introduction

All aerobic organisms require oxygen for survival. In the human body, oxygen from the lungs is transported by red blood cells to the mitochondria, where it is used during cell respiration to generate energy. Imaging methods to visualize cellular metabolism and biological functions, such as blood oxygen levels and pH levels, are being developed for the treatment of certain diseases, such as cancer [1,2,3]. The amount of dissolved oxygen (DO) in blood is evaluated in terms of partial pressure of oxygen (pO_2_), which is an important clinical medical parameter. Although blood oxygen concentration is important, the direct measurement of DO changes in tissue fluid around cells or tissues caused by metabolism may provide a more detailed picture of metabolic activity. Multiple methods for measuring DO concentrations have been reported. The standard method for assessing DO concentration in tissues is the polarographic electrode method [4]. Surface DO can be measured using a multiwire electrode, and needle-shaped microelectrodes are used for measurements within a tissue [5,6,7]. However, using this method, DO concentrations can be measured only at those localized points where the electrode is placed. Spatial measurements can only be obtained by repeating the measurements multiple times over a certain area. Consequently, this method is invasive and there is a risk of tissue damage. An ion-sensitive field-effect transistor (ISFET)-based DO sensor was reported by Sohn in 1996 [8]. However, similar to the methods listed above, the device employed amperometry, which measures oxygen concentration indirectly through the complex reaction of oxygen reduction. In in vivo measurements, the series of reactions necessary for DO sensing may be interfered by biological homeostasis, and sensing consumes oxygen, which may invasively damage tissues. Therefore, this amperometric measurement is not suitable for in vivo measurements. Therefore, we believe that it is more advantageous if DO can be detected potentiometrically, which can sense the oxygen absorbed on the sensor surface without oxygen consuming, resembling the detection principle of hydrogen ions (H^+^) in an ISFET and ISFET technology-based sensors [9,10].

**Figure 1:**
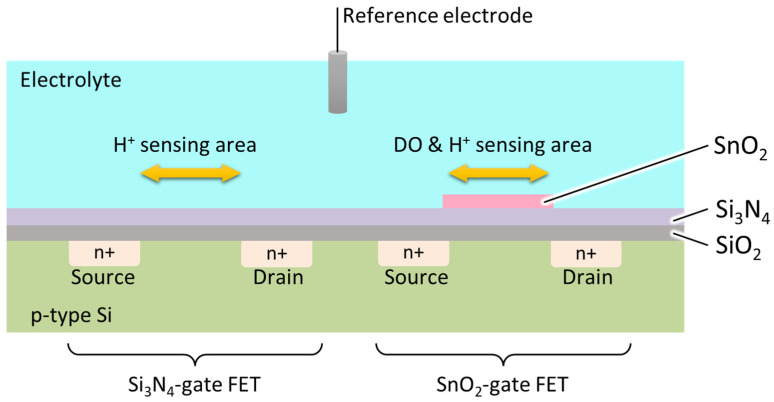
Schematic diagram of the proposed DO-/hydrogen ion-sensitive FET.

In this study, we propose a potentiometric DO-sensing device based on a field-effect transistor (FET). We used SnO_2_, which is an oxygen-sensitive material widely used in oxygen gas sensors and is seemingly used as a hydrogen ion-sensing film in ISFETs [11,12,13,14]. However, SnO_2_ is expected to respond to a variety of ions similarly to other metal oxides. Therefore, we propose a multimodal sensing approach to compensate for the insufficient selectivity and specificity by combining multiple sensors. As a first step in the development of the DO-sensing device based on the multimodal sensing concept, in this study, we focused on pH correction because it is expected that SnO_2_ will respond to both hydrogen ions and DO. We propose a device for the monolithic integration of a hydrogen ion-sensitive FET and a DO-sensitive FET onto a single chip, as shown in Figure 1. This is because the silicon nitride (Si_3_N_4_) thin film has ion selectivity only towards hydrogen ions. From the perspective of the multimodal sensing approach, selecting a material for the sensing membrane that can be formed by the CMOS compatible process is important for the monolithic integration of multiple sensors, and both SnO_2_ and Si_3_N_4_ satisfy the CMOS compatibility requirements. By simultaneously measuring the hydrogen ion concentration of the solution, that is, the pH, using an integrated hydrogen ion-sensitive FET, the hydrogen ion-induced output component of the SnO_2_-gate FET can be eliminated, and an accurate DO can be obtained. In this study, we fabricated a multimodal sensing device by monolithic integration of FETs with SnO_2_ and Si_3_N_4_ as the sensitive layers and measured its response to pH and different DO concentrations in solutions.

## 2. Materials and Methods

### 2.1. Principle of H^+^ and O_2_ Sensing

Most ion sensors are potentiometric sensors, detecting ions by measuring the electrical potential difference Δ∅ at a solid/liquid interface as a function of the ion concentration to be determined. In a hydrogen ion-sensitive FET which has a Si_3_N_4_/SiO_2_/Si structure as the gate area, the Δ∅ between the electrolyte and the Si_3_N_4_ layer is given by the Nernst equation [15]:

(1) $$\Delta \emptyset =V_{REF}+\frac{RT}{nF}\ln\alpha_{H^{+}}$$

where *V_REF_* is the reference electrode voltage, *R* is the gas constant, *T* is the absolute temperature, *n* is the number of moles of electrons transferred in the reaction, and *F* is the Faraday constant. Because $\alpha_{H^{+}}\;$is the H^+^ concentration, it can be calculated using the measured Δ∅ at constant *V_REF_* and constant *T*.

Metal oxide gas sensors are among the most studied gas sensors. Although the nature of solid interactions is poorly understood, in most studies, the principle of detection is based on conductivity change, or changes in the capacitance, work function, mass, or optical characteristics of the gas-sensing metal oxide material [16,17]. Most gas sensors utilize semiconductor materials such as zinc oxide (ZnO) and SnO_2_, and they are believed to operate via the adsorption of oxygen on the surface owing to oxygen vacancies, leading to a high resistance. Studies have also been conducted on FET-based gas sensors. The first hydrogen-sensitive gas FET (Lundström-FET) was introduced by Lundström in 1975, whereby a thin palladium (Pd) layer was formed as a gate electrode on an ordinary metal oxide semiconductor field-effect transistor [18]. In this device, hydrogen gas modifies the work function of Pd, which changes the threshold voltage of the transistor. The suspended gate FET (SG-FET) was introduced by Lorenz in 1990 for gas sensing [19]. The idea behind this device is to measure the work function change due to gas adsorption on its sensing film via an FET. Using this method, a variety of gases can be detected by replacing the sensitive layer material.

In our study, we are interested in adopting the gas-FET and SG-FET principles and applying them to the detection of oxygen gas in liquid media. SnO_2_ is a widely studied gas-sensing material, and its interaction with oxygen has been reported [14,20]. In our proposed device, the adsorption of oxygen to the oxygen vacancies on the surface of SnO_2_ modifies the work function of the material, which leads to a change in the threshold voltage of the transistor. We would also like to point out that we chose SnO_2_ because it is compatible with the fabrication process based on a standard CMOS. The similarity in the structures of the gas-FET and hydrogen-sensitive FET is also convenient for the development of pH and DO multimodal sensors.

### 2.2. Sensor Design

The proposed device, following the conventional structure of an ISFET, consists of an ion-sensitive layer/SiO_2_/Si structure, whereby the ion-sensitive layer is Si_3_N_4_ for hydrogen ion sensing and SnO_2_ for DO sensing. Both the SnO_2_-gate and Si_3_N_4_-gate FETs were designed to have a channel width (*W*) of 800 µm. Two different channel length (*L*) devices were designed: 100 µm and 200 µm, giving aspect ratios (*W*/*L*) of 8 and 4, respectively. The *W*/*L* ratio controls the transconductance ($g_{m}$) of the FET, as shown in Equation (2); the higher the $g_{m}$, the higher the drain-source current change produced by a gate-source voltage change at a fixed drain-source voltage. In FET devices, high $g_{m}$ is ideal for utilizing the full capability of the linear region as a transduction element. The sensitivity of the FET to either DO or hydrogen ions is determined by the material of the gate dielectric and is not influenced by its aspect ratio.

(2) $$g_{m}={\frac{\partial I_{DS}}{\partial V_{GS}}|}_{V_{DS}=const}$$

The geometrical design of the FETs follows that of a conventional ISFET. However, packaging constraints are considered when designing the chip layout. As the FETs were immersed in the solutions, the source/drain diffusion area was extended, terminating in small contact holes at the ends. This increases the distance of the sensing/gate region from the metal contact pads, making it easier to apply the epoxy coating for waterproofing.

### 2.3. Sensor Fabrication

**Figure 2:**
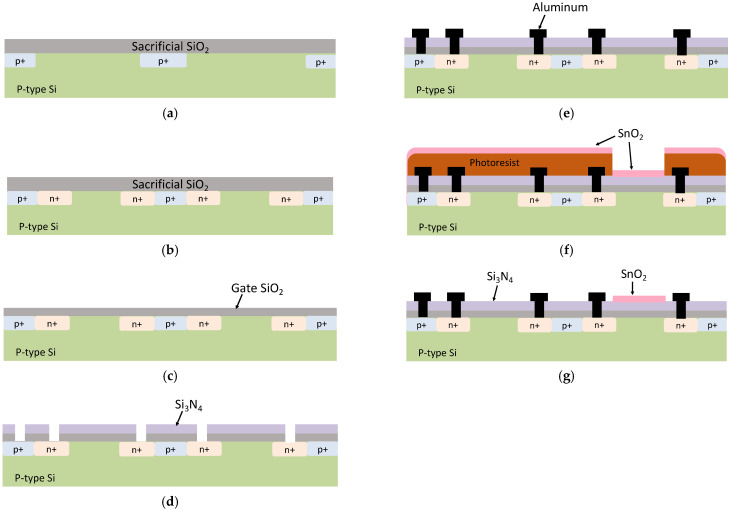
Cross-section diagram showing the fabrication process of the proposed device. (**a**) Sacrificial oxide formation and channel stopper ion implantation. (**b**) Source/drain ion implantation. (**c**) A new layer of gate oxide (SiO_2_) is formed. (**d**) Deposition of Si_3_N_4_, and source/drain contact opening. (**e**) Aluminum contact sputtering. (**f**) Deposition of SnO_2_ on top of a photoresist mask. (**g**) Lift-off of photoresist mask.

A 4-inch p-type silicon wafer was used as the starting material. The fabrication of the FETs was based on the standard CMOS process, and the steps are shown in Figure 2 and are as follows:

- A sacrificial SiO_2_ layer was formed on the p-type silicon wafer, and p^+^ channel stoppers were formed via ion implantation.
- Source/drain ion implantation was carried out.
- During implantation, damage may be induced in the gate oxide layer [21,22]; therefore, a new SiO_2_-gate oxide layer (70 nm) was formed via wet oxidation.
- The hydrogen ion-sensitive layer, Si_3_N_4_ (130 nm), was deposited using low-pressure chemical vapor deposition (LP-CVD). The source/drain contact holes were then opened.
- Aluminum (1 µm) was deposited using DC sputtering at 1000 W, 0.5 Pa of Ar.
- A photoresist mask was applied, and the DO-sensitive layer, SnO_2_ (100 nm), was deposited by sputtering at 300 W, using a gas mixture of 90% Ar and 10% O_2_.
- Using the lift-off method, SnO_2_ was patterned on top of the gate of a DO-sensitive FET. O_2_ gas annealing was performed at 440 °C for 30 min.

**Figure 3:**
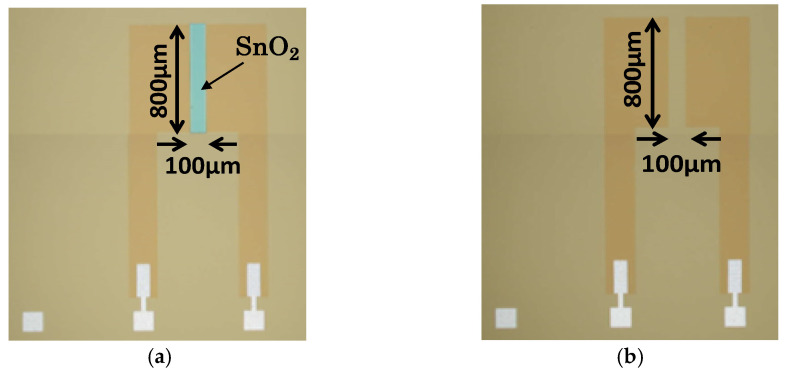
Optical microscope photo of the fabricated FET. (**a**) SnO_2_-gate FET; (**b**) Si_3_N_4_-gate FET.

An optical microscopy image of the fabricated device is shown in Figure 3. The devices were encapsulated using an epoxy resin, leaving only the gates (sensing regions) exposed to the test solution. The responses of the SnO_2_-gate FET and Si_3_N_4_-gate FET to DO and hydrogen ions were investigated based on the drain current–gate voltage (I_d_–V_gs_) characteristics.

### 2.4. Measurement Procedure

**Figure 4:**
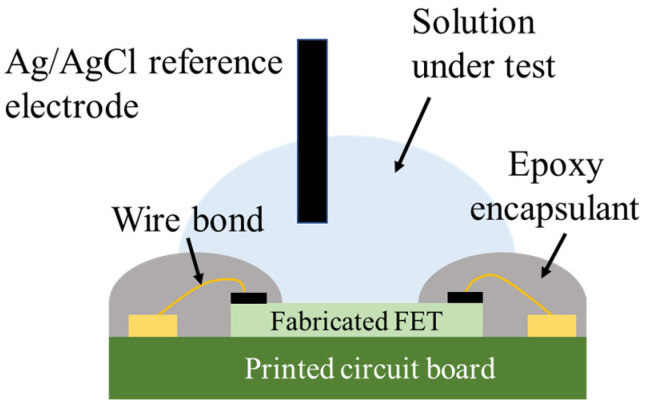
Schematic illustration of the cross-section of the device during measurement.

**Figure 5:**
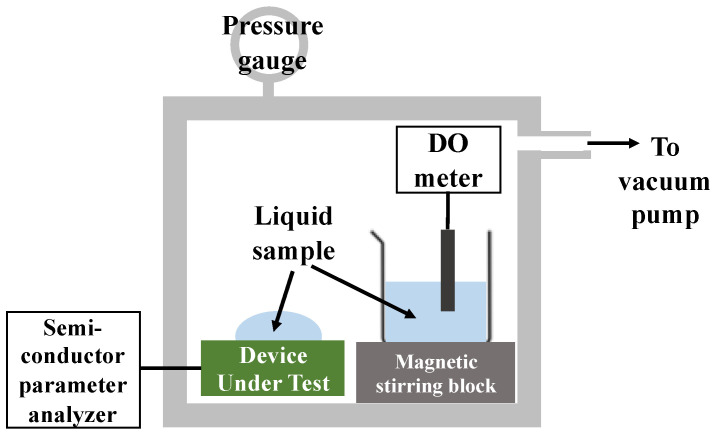
Schematic illustration of the experimental setup for DO measurement.

An external silver/silver (I) chloride (Ag/AgCl) glass reference electrode was suspended above the FET to ensure that the bottom edge touched the solution surface (Figure 4). The source, drain, and reference electrodes were then connected to a semiconductor parameter analyzer (Agilent Technologies B1500A, Santa Clara, CA, USA) for the I–V measurements. Both the SnO_2_-gate FET and Si_3_N_4_-gate FET were operated in constant drain voltage (V_ds_ = 50 mV) mode, and the source and bulk were ground. Buffer solutions with pH values of 4.01, 6.86, and 9.18 were used for the pH measurement. To determine the DO response of the FET without any influence from pH—that is, hydrogen ion changes in the test solutions—the DO concentration of the solution under test was controlled by deoxygenation through degassing. In this method, 100 µL of pH 6.86 standard buffer solution was placed onto the sensing area of the FET, which was placed inside a chamber containing another beaker of the same solution and a DO meter (Edge DO Meter HI 2040-01 HANNA Instruments, Woonsocket, RI, USA) inserted into the beaker (as shown in Figure 5). The air inside the chamber was pumped out using a vacuum pump and the DO concentration of the solution was monitored using a DO meter. The oxygen concentration of the liquid decreases proportionally with the air pressure inside the chamber. When the solution reached the desired DO concentration, the pump was stopped and the measurement was performed. Note that as a preliminary experiment, we verified that the pH of the solution does not change due to changes in DO caused by vacuuming.

## 3. Results and Discussion

### 3.1. Electrical Characterization Results

**Figure 6:**
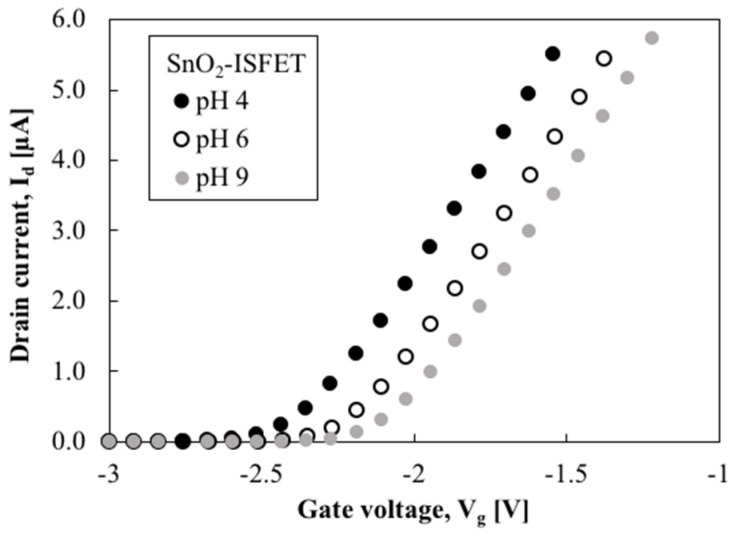
I_d_–V_g_ of SnO_2_-gate FET in various pH solutions.

**Figure 7:**
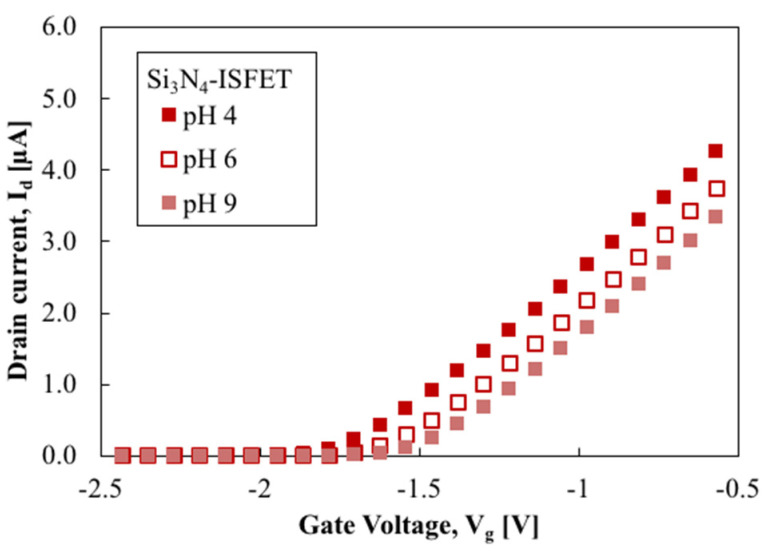
I_d_–V_g_ of Si_3_N_4_-gate ISFET in various pH solutions.

Figure 6 and Figure 7 show the drain current, I_d_, versus the gate voltage, V_g_, of the SnO_2_-gate FET and Si_3_N_4_-gate FET, respectively. This electrical characterization shows that both FETs have the typical characteristics of field-effect transistors. No hysteresis effect was observed in the SnO_2_-gate FET when positive and negative sweeps were applied. Note that the $g_{m}$ of the Si_3_N_4_-gate FET was much smaller than that of the SnO_2_-gate FET. This is because the gate length of the Si_3_N_4_-gate FET was 100 µm while the gate length of the SnO_2_-gate ISFET was 200 µm. The I_d_–V_g_ results show that the current is proportional to the applied voltage, which agrees with the characteristics of FET, whereby the curve shifts to the left in low pH conditions and shifts to the right in high pH conditions. Repeated cycling of the sensors between various pH buffer solutions was carried out by rinsing with deionized water and then applying different pH solutions. In all cases, the sensors recovered their original values, confirming reproducibility.

### 3.2. Sensitivity of the FET Device

To discuss the sensitivity of the FET, the sensing output voltage (V_out_) was determined as the V_g_ of the FET at I_d_ = 1 µA of I_d_–V_g_ characteristics, as shown in Figure 6 and Figure 7. When I_d_ and V_d_ were kept constant, the changes in the threshold voltage induced by the changes in the pH of the test solution caused identical changes in the gate voltage. Thus, the pH sensitivity of the FET can be expressed using the following equation:

(3) $$S_{\mathrm{pH}}={\left|\frac{\partial V_{g}}{\partial \mathrm{pH}}\right|}_{I_{d}\;const}$$

**Figure 8:**
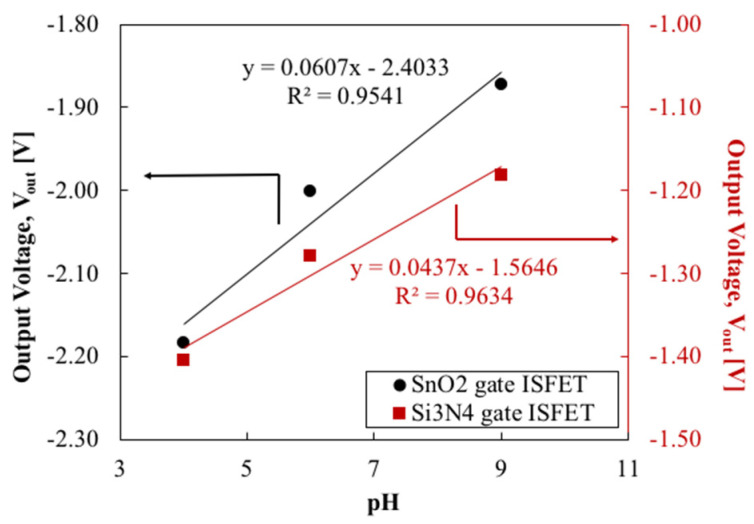
Output voltage of FET in response to pH change.

The change in V_out_ due to the change in pH is plotted in Figure 8. The SnO_2_-gate FET exhibited a pH sensitivity of 60.7 mV/pH and the Si_3_N_4_-gate FET exhibited a pH sensitivity of 43.7 mV/pH. The sensitivity of the Si_3_N_4_-gate FET is in line with the reported sensitivity [11] and obeys the Nernstian limit. However, the SnO_2_-gate FET exhibits a slightly super-Nernstian pH response, which is common in metal oxide-based pH sensors [23]. This may be due to the oxygen content of the solution and the structure of the oxide. Both FETs showed a satisfactory linear response to the pH. As for the pH measurement range, SnO_2_-gate FETs have been reported to demonstrate a measurement range of pH 2–10 [11], and Si_3_N_4_-gate FETs have been known to exhibit a suitable Nernstian response from pH 2 to 12 [24]. Similar characteristics can be expected for the fabricated devices, which have the same basic structure and use the same sensing material.

Dissolved oxygen (DO) is basically non-compound oxygen molecules dissolved in water. Therefore, for the DO response, it is hypothesized that oxygen molecules in the test solution adsorb onto the oxygen-vacant surface of the SnO_2_-sensitive layer, thereby modifying its work function, which then induces a change in the threshold voltage. This causes an identical change in gate voltage. Thus, the DO sensitivity of the FET can be expressed using the following equation:

(4) $$S_{\mathrm{DO}}={\left|\frac{\partial V_{g}}{\partial \mathrm{DO}}\right|}_{I_{d}\;const}$$

**Figure 9:**
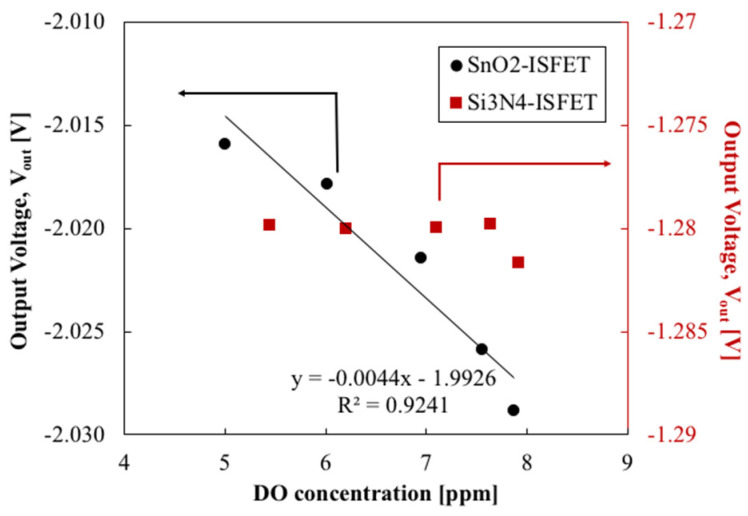
Output voltage of FET in response to DO change.

The change in V_g_ owing to the change in DO is plotted in Figure 9. The SnO_2_-gate FET exhibited a DO response with a sensitivity of 4 mV/ppm. V_out_ of the SnO_2_-gate FET decreased with increasing DO concentration. This response was not observed for the Si_3_N_4_-gate FET. These findings suggest that the SnO_2_ layer is DO-sensitive, whereas the Si_3_N_4_ layer is not. In this evaluation, the lowest measured DO was 5 ppm. If we assume that the FET response is linear to the amount of DO, the output voltage of the SnO_2_-gate FET at 0 ppm is expected to be −1.99 V, as shown in Figure 9. This is considered an acceptable value based on the characteristics of the FET as an electronic device. Although the details of the oxygen detection mechanism should be discussed separately, if the FET threshold is shifted in the positive direction because of oxygen adsorption on the sensing membrane surface, as assumed in Section 2.1, it may be possible to detect the absence of adsorbed oxygen, i.e., 0 ppm DO. As for the upper limit of measurement, the DO of the solution reaches equilibrium at approximately 8 ppm for air at room temperature and atmospheric pressure, which corresponds to the maximum DO value of the solution measured in this study. It is expected that even higher DO concentrations may be measured, but oxygen adsorption on the membrane surface will eventually saturate. A detailed evaluation of the lower and upper limits of DO measurement will require additional verification through an investigation based on an experimental system that allows a wider range of DO variation.

Because SnO_2_ is sensitive to both pH and DO, in conditions where the solution undergoes both pH and DO changes, it is not possible to measure them simultaneously using a discrete SnO_2_-gate FET. As the sensor output voltage is a function of two parameters, pH and DO, it can be written as follows:

∆V_out_ = *f* (∆pH, ∆DO) (5)

where ∆V_out_ is the change in the output voltage, ∆pH is the change in pH, and ∆DO is the change in DO. As the simplest example, two independent parameters can be expressed as a linear combination using the following equation.

∆V_out_
*= g* (∆pH) + *h* (∆DO) (6)

If the pH and DO responses follow Nernst’s equation, the equation can be transformed as

∆V_out_ = *S*_pH_*⋅*∆pH *+ S*_DO_*⋅*∆DO (7)

where *S*_pH_ is the pH sensitivity and *S*_DO_ is the DO sensitivity. Hence, when integrated with a hydrogen ion-sensitive FET, ∆DO can be obtained as ∆pH is known. Simultaneous sensing of DO and pH can be achieved by multimodal sensing using a SnO_2_-gate FET and Si_3_N_4_-gate FET. Because both pH and DO sensing principles involve adsorption and desorption on the sensing membrane, and the total amount of ions and oxygen adsorbed on the membrane could affect the output, it is ideal to treat the sensor output as the simplest linear combination. On the other hand, it should be noted that the independence and coherence of the two parameters have not yet been verified, and thus, a detailed verification under various measurement conditions is required.

There are examples of pH-ISFET-based sensors related to CMOS-compatible DO sensing devices [8,25]. However, these devices are based on an irreversible detection principle that consumes DO during sensing operations. On the other hand, our device is based on the reversible phenomenon of oxygen adsorption/desorption on the sensing membrane, and it does not interfere with the measurement target. A sensor based on FETs has also been developed as a multi-ion sensing device [26,27,28]. The DO-sensing device in this study is compatible with various multi-ion measurement FET sensors that have been developed in the past. We have realized a new sensing modality in the field of FET-type sensors via the addition of DO. We expect that this can be further expanded to multi-ion and DO-sensing devices.

## 4. Conclusions

A simple device combining SnO_2_-gate and Si_3_N_4_-gate FETs has been presented. The fabricated device was characterized, and the results suggest that the proposed sensor, when operated in combination with FETs, is feasible for the determination of DO concentration and pH. The results imply a useful application of solid-state FET devices for the detection of DO concentrations in liquid media. Moreover, because FET-based potentiometric sensors are compatible for array construction, a multimodal sensing array of DO and pH can be expected. Sophisticated sensing arrays are novel tools in biomedical applications.
